# Notes and News

**Published:** 1892-12-31

**Authors:** 


### Notes and News.

Collected and Communicated.

It is with very sincere regret that we have to record the death of Mrs. Wardroper, who was appointed Matron to St. Thomas's Hospital so long ago as 1854. Upon Mrs. Wardroper devolved the responsible duty of creating and building up a system of trained nursing upon practical lines, which should eliminate all the evils of the days of which Dickens has so graphically written, and at the same time be comprehensive enough to meet the growing demands in nursing matters of medical science and a quickened public intelligence. A memoir by Miss Florence Nightingale will be found in this week's Nursing Supplement.

A meeting of the Council of the London and Counties Medical Protection Society was held at Hatter Street, Bury St. Edmunds, on Wednesday, December 21st, Dr. Fegen, of Brandon, presiding. After some general business had been transacted, it was decided to hold a general meeting of the members and others interested in medical protection at Bury St. Edmunds at an early date in 1893, Mr. John Kilner, F.R.C.S., Senior Surgeon to the West Suffolk Hospital, being invited to preside. Dr. Mead (Newmarket), the Organising Secretary, reported that at least thirty divisions had been already formed, and that at a recent meeting of the Council 142 new members had been elected.

A nursing home, intended to fulfil a new and useful purpose, is now open in Devonshire Street. The movement owes its origin to the Church House, and if the difficulties of the undertaking can be surmounted successfully it will prove a great blessing to the poorer clergy and missionaries especially. Payments are required according to circumstances, and free treatment will be provided in special cases. The movement is already supported by eleven bishops, who have become vice-presidents, and a large staff of eminent physicians and surgeons have proffered honorary services. Those who wish to receive further information or to assist this excellent work should write to Canon Cooper, The Church House, Westminster, who is acting as honorary secretary.

It is well that those who are in the habit of taking antipyrine frequently and in large doses to know that such practices are not always indulged in with impunity. During the cholera scare a patient was admitted into the Moabit Hospital, in Berlin, supposed to be suffering from an attack of cholera. The symptoms proving unusual, inquiry elicited that the sufferer was in the habit of taking antipyrine daily as a cure for headache, steadily increasing the dose, until seized with symptoms resembling those of cholera. Dr. Guttmann records that he has met with many cases of poisoning by antipyrine during the past five years; it cannot, therefore, be taken without caution as many suppose.

The unqualified assistant is a danger amongst druggists in France, as well as in England, it appears. Quite recently the prisoners in the Beauvais prison exhibited signs of a violent malady which was at first supposed to be cholera. An investigation was made by the doctor, who suspecting the drinking water, with which was mixed an extract of walnut leaves, drank some himself. He was almost immediately seized with violent symptoms of poisoning. Prompt measures were taken, however, and the doctor recovered shortly. On inquiry, it proved that the chemist employed, being absent, his pupil had supplied belladonna in lieu of extract of walnut leaves. It would seem that the French Courts have a different understanding of where the guilt lies in such a case from our presiding judges, and Mr. François, the chemist, and not his assistant, received punishment.

Where will the responsibilities and undertakings of the School Board end? Pianos have been favourably received, and now another idea, of a more definitely salutary nature, we must admit, is gaining ground. This is the regular supervision of the children's teeth by a competent dentist. Teeth are, no doubt, both important and essential elements in the well-being of the whole community, and regular visits to the dentist would be of advantage to rich and poor alike; but still we cannot help thinking there should be limits to the Education Act liabilities. Free dinners to poor children form part of a voluntary system, which leaves those who approve, or disapprove, to act according to their opinions. But if the ratepayer is bound, despite his principles and convictions, to remove all manner of responsibility from off the parents' hands at his own expense, the paupers thus manufactured will be in so enviable a condition, that all incentive to honest labour will be removed. Metropolitan School Boards are now asked to provide dental supervision for the scholars attending their schools, at a cost to each district of at least £150 per annum, and £40 for initial expenses. This, in spite of the excellent opportunities provided for the poor at Guy's Hospital, and many other like institutions, which not only benefit the poor directly, but indirectly also, by providing means of instruction under skilled superintendence to a large number of students. If dentistry is to be made a special feature in the advantages offered by the School Board, we imagine the advocates of good food and clothing as essentials to health, will not be backward in putting in a plea for a free drapery and provision store to be attached to every school.

